# Lens Endogenous Peptide αA66-80 Generates Hydrogen Peroxide and Induces Cell Apoptosis

**DOI:** 10.14336/AD.2016.0805

**Published:** 2017-02-01

**Authors:** Murugesan Raju, Puttur Santhoshkumar, K. Krishna Sharma

**Affiliations:** ^1^Departments of Ophthalmology and; ^2^Biochemistry, University of Missouri School of Medicine, Columbia, MO65212, USA

**Keywords:** crystallin, peptide, hydrogen peroxide, lens, cataract, amyloid

## Abstract

In previous studies, we reported the presence of a large number of low-molecular-weight (LMW) peptides in aged and cataract human lens tissues. Among the LMW peptides, a peptide derived from αA-crystallin, αA66-80, was found in higher concentration in aged and cataract lenses. Additional characterization of the αA66-80 peptide showed beta sheet signature, and it formed well-defined unbranched fibrils. Further experimental data showed that αA66-80 peptide binds α-crystallin, impairs its chaperone function, and attracts additional crystallin proteins to the peptide α-crystallin complex, leading to the formation of larger light scattering aggregates. It is well established that Aβ peptide exhibits cell toxicity by the generation of hydrogen peroxide. The αA66-80 peptide shares the principal properties of Aβ peptide. Therefore, the present study was undertaken to determine whether the fibril-forming peptide αA66-80 has the ability to generate hydrogen peroxide. The results show that the αA66-80 peptide generates hydrogen peroxide, in the amount of 1.2 nM H_2_O_2_ per µg of αA66-80 peptide by incubation at 37°C for 4h. We also observed cytotoxicity and apoptotic cell death in αA66-80 peptide-transduced Cos7 cells. As evident, we found more TUNEL-positive cells in αA66-80 peptide transduced Cos7 cells than in control cells, suggesting peptide-mediated cell apoptosis. Additional immunohistochemistry analysis showed the active form of caspase-3, suggesting activation of the caspase-dependent pathway during peptide-induced cell apoptosis. These results confirm that the αA66-80 peptide generates hydrogen peroxide and promotes hydrogen peroxide-mediated cell apoptosis.

The human eye is one of the superior visual systems [1]. The high refractive power of its built-in lens, located in the anterior part of eye, focuses the image of the visual field on the retina [2]. It is well documented that the focusing ability of the lens and lens transparency are reduced with aging. Age-related changes of visual range and refractive power can begin as early as the fourth decade of life [3, 4]. Age-related lens opacification is a leading cause of blindness across the globe and highly prevalent in the elderly population. Global data as well as national data reveal that vision impairment and blindness have increased 23 percent since the year 2000 and contribute to significant increase in the medical care expenditure toward vision care [5].

The lens tissue is mainly composed of long-living crystallin proteins, classified as α-, β- and γ-crystallins, based on their structure and genetic organization [6]. α-Crystallin is composed of two 20kD subunits, αA and αB. Both αA- and αB- function like chaperone molecules and are believed to be important for lens transparency [7, 8]. During the aging process, lens crystallins slowly lose chaperone function and, with time, are depleted from the soluble form in the lens nuclear region, leading to lens opacity and decreased visual acuity [9]. Chromatographic profiles of lens extracts show that the amount of water-soluble α-crystallin is negligible in the nuclear region of aged lenses, with the bulk of the α-crystallin partitioned into water-insoluble aggregates [10-12]. Biochemical and biophysical analyses of aged lens tissue show an accumulation of post-translationally modified crystallins [13-15], fragmented proteins [16, 17] and low molecular weight (LMW) peptides [18, 19] as compared to young lens tissue. We have also identified several LMW peptides in an animal model of cataract [20]. We and other investigators have reported the occurrence of LMW peptides in human lenses and have found a correlation between lens aging and increasing amounts of LMW peptides [19, 21]. Further, we have also established by in vitro studies a strong correlation between the amount of of LMW peptides and the formation of larger light scattering aggregates [18]. Peptides derived from α-crystallin were predominated in aging lens [19]. Of the α-crystallin-derived peptides, αA66-80 peptide and its derivatives originating from β3-β4 strands of native α-crystallin were present in significantly higher concentrations than other peptides. The αA66-80 peptide and its derivatives are capable of forming fibrils under physiological conditions [18], similar to Aβ peptide [22, 23].

Oxidation is a key mechanistic factor in many pathological conditions, including age-related cataract. The primary evidence for oxidation-mediated lens opacity originated from the clinical observation, reported 30 years ago, that patients receiving hyperbaric oxygen (HBO) therapy develop lens opacities similar to nuclear cataract [24]. In 1995 Giblin validated this clinical observation in a laboratory setting by demonstrating that the HBO-treated guinea pig lens exhibits pathology similar to that observed in human aged and cataractous lens tissues, including loss of soluble protein, excessive disulfide crosslinking and larger light scattering aggregates [25]. The nuclear opacity induced by HBO in guinea pig lens continues to serve as the closest available animal model of human age-related cataract. Adding to the evidence of the role of oxidation in cataract formation were the in vitro studies by Ortwerth and colleagues, who demonstrated that oxidation leads to the formation of larger light scattering aggregates in lens proteins [26, 27]. These studies, along with other reports [28, 29], established a strong link between oxidation and the formation of high molecular weight aggregates of lens proteins [25]. The key ingredients for oxidation are oxygen and metal ion. However, unlike the brain and other tissues, the lens is a unique avascular tissue with low oxygen [30]. Hence, the lens was long considered to be an “oxygen tight can,” until optical oxygen sensors became available for measurement of oxygen levels in the lens. In 2006 Beebe and colleagues used an optical oxygen sensor to measure oxygen levels in the eyes of rabbits breathing different oxygen concentrations and found that oxygen consumption increased in the posterior region of the lens with increasing concentrations of inspired oxygen [31]. The dissolved oxygen levels normally found in the vitreous of eye have been reported to be varying from species to species [31, 32]. Other investigators measured the soluble oxygen level in the central bovine lens, using a fiberoptic probe detection system, and found that the lens has 1.6 ± 0.5 mm Hg oxygen [30]. Further, the presence of metal ions, including Cu (II) and Zn (II), is well demonstrated in lens tissues [33, 34]. We found that the αA66-80 peptide has a Cu (II) binding capacity [35]. Therefore, we hypothesized that the trace amount of metal ions and the dissolved oxygen in the lens tissues would be sufficient for redox activity display by the fibril-forming αA66-80 peptide. Furthermore, the Aβ peptide has been shown not only to form fibrillar assembly but also to exert cytotoxicity by generating hydrogen peroxide (H_2_O_2_) [36]. Therefore, we undertook the present study to test whether the fibril-forming αA66-80 peptide generates a hydrogen peroxide-mediated cytotoxic effect. We found that the αA66-80 peptide generates H_2_O_2_ and induces cell apoptosis under culture conditions.

## MATERIALS AND METHODS

### Materials

Peptides used in this study were synthesized and purified for more than 95% homogeneity by the manufacturer. The peptides αA66-80 [SDRDKFVIFLDVKHF], proline-substituted αA66-80 (V72P) [SDRDKFPIFLDVKHF] and alanine-substituted αA66-80 (H79A) [SDRDKFPIF LDVKAF] were supplied by GenScript (Piscataway, NJ). The β-amyloid peptide was procured from EZBiolab (Westfield, IN). Peptide stock solutions were prepared freshly before each experiment, at 2 mg/mL concentration in sterile water. For the H_2_O_2_ assay, the stock peptide solution was diluted, in a 1:1 ratio, in phosphate buffer (50 mM PO4 + 150 mM NaCl, pH 7.2) containing glycine (1 mM), unless otherwise specified.

### Isolation of LMW peptides from aged and young human lens tissues

Human lenses obtained from the Heartland Lions Eye Bank (Columbia, MO) were used to isolate LMW peptides, using the procedure described earlier [19]. In brief, 73-year-old lenses, 43-year-old lenses and young 17-year-old lenses were homogenized in phosphate buffer (50 mM, 150 mM NaCl, pH 7.4) containing 6 M urea and the reducing agent dithiothreitol (10mM DTT). The homogenate was centrifuged at 16,000 × g for 1 h and urea-soluble supernatant was passed through a 10 kDa cut off membrane filter (Millipore) to obtain LMW peptides. The 10 kDa cutoff filtrates of aged (70-year-old), middle (43- year-old) and young (17-year-old) lenses were desalted using Supelco supelclean LC-18 spin columns (Sigma, St louis, MO). The bound peptides were eluted by 70% acetonitrile and dried in a speedvac system. The dried peptides were weighed and dissolved in sterile water and used (0.1mg/0.1mL) in the experiments.

### Hydrogen peroxide assay

Amplex Red Hydrogen Peroxide assay kit was purchased from Molecular Probe (Eugene, OR), and the assay was performed as per the manufacture’s protocol. For the hydrogen peroxide assay, peptide stock solution (50 µl) was diluted to100 µl with phosphate buffer, pH 7.2, and incubated at 37°C. At the end of incubation, Amplex Red reagent was added and the mixtures were further incubated for 30 min in the dark at room temperature. The fluorescent intensities of the reaction mixtures were measured by exciting the samples at 530 nm (bandwidth 5 nm), and the emission intensities were recorded at 590 nm (bandwidth 5 nm), using a Jasco spectrofluorimeter FP-750. The average fluorescence emission intensity was calculated from three independent experiments. The arbitrary FU was converted to nano molar scale using standard graph prepared with 1 nM to 1 mM of know amount of H_2_O_2_.

### Analysis of copper binding to αA66-80 and mutant αA66-80 peptide (αA66-80 H79A) by mass spectrometry

The mass spectrometric analysis of the peptides treated with excess copper was carried out at the University of Missouri Proteomics Center on an Agilent 6520 QTOF mass spectrometer. The method used to determine copper binding to peptides described earlier [35] was followed in these experiments.

### Purification of crystallin proteins and preparation of tryptic digest

Ten human lenses (43-45 years old) were homogenized in phosphate buffer (pH 7.2) containing reducing agents and protease inhibitor mixture (Sigma-Aldrich, St. Louis, MO). The sample was centrifuged at 16,000 × g for 1 h. The water-soluble fraction was collected and different crystallin fractions were separated by gel filtration on a Sephadex G-200 column. Protein concentration was measured using Bio-Rad protein assay method. α-, βH-, βL- and γ-crystallins (3 mg each) were individually digested with sequence-grade modified trypsin in 50 mM phosphate buffer (pH 7.4). The tryptic digestion was performed at 37°C for 18 h, using 1:100 ratio (wt/wt) of enzyme to protein. At the end of trypsin digestion, the enzyme-digested samples were passed through 10 kDa cut-off membrane filter and the filtrates were quantified by micro BCA method, 100 µg peptide was directly used for H_2_O_2_ assay in phosphate buffer.

### Cell culture experiments

Cos-7 cells were cultured in Dulbecco’s modified Eagle’s medium (DMEM), supplemented with 10% fetal bovine serum, 100 μg/mL streptomycin and 100 units/mL penicillin, at 37 °C in an incubator with a 5% CO_2_ atmosphere. Cells were seeded at the density of ~25000 cells/cm^2^ in an 8-well slide chamber and allowed to grow for 24 h. After 80% confluency, the cells were treated with αA66-80 peptide (0.1mg/mL) in 0.5 mL of serum-free medium. Wells treated with proline-substituted αA66-80, (0.1mg/mL) and the serum-free medium served as controls. The cells were incubated at 37°C cells until the apoptosis assays were performed

### TUNEL assay

Terminal deoxynucleotidyl transferase dUTP nick end labeling (TUNEL) assays were performed 24 h after treating the cells with the peptides using ApopTag Red In Situ Apoptosis Detection Kit (Chemicon, Temecula, CA). In brief, αA66-80 peptide-transduced cells were fixed in 4% paraformaldehyde overnight at 4°C. Fixed cells were washed twice with phosphate-buffered saline for 5 min. For TUNEL reagent permeability, cells were post fixed in an ethanol/acetic acid mixture (2:1). TUNEL incorporated 3′ end nucleotide labeling was detected using an anti-digoxigenin-rhodamine solution with counter stain DAPI (4-,6-diamidino-1-phenylindole). A fluorescence micro-scope (Leica DMR) was used to observe positive cells, and the images were recorded using an Optonics digital camera. The percentage of apoptotic cells was calculated by counting TUNEL-positive cells divided by the total number of cells visualized in a given area.

### Expression of cleaved caspase-3

Apoptosis is a programmed cell death activated by a cascade of caspase enzymes. Expression of cleaved caspase-3 is a marker for the activation of the apoptotic signalling pathway. To measure cleaved caspase-3, Cos-7 cells treated with peptides for 48 h were fixed in 4% paraformaldehyde in PBS for 20 min. The cells were washed twice in PBS, permeabilized with 0.1% Triton X-100 for 5 min, blocked for 1 h with 1% BSA in PBS before adding rabbit cleaved caspase-3 antibody (Cell Signalling, Danvers, MA) (1: 400). The cells were washed with PBS and incubated with secondary antibody anti-rabbit IgG (1:300) + DAPI (1:300) for 1hr at RT covered from light. The cells were washed and observed under fluorescent microscope for cells expressing cleaved caspase-3.

### Entry of aA66-80 peptide into cells

To test the entry of peptide in to the cells, we have used Fluorescein isothiocyanate (FITC) conjugated (C-terminal) αA66-80 peptide (αA66-80-FITC) and primary cultures of pig lens epithelial cells (LEC). Epithelial cells were collected during the pig lens epithelial explant cultures we prepared for another study (37). LECs (1 x10^4^) grown overnight on an 8-well chamber slide was treated with αA66-80-FITC peptide (10 µg/mL) in serum-free DMEM, in a final volume of 0.5 mL, for 24 h at 37°C and 5% CO_2_. At the end of incubation, the cells were washed twice in PBS, fixed in 4% paraformaldehyde for 20 min, stained with DAPI (1:300) + Phalloidin (1:2000) in PBS for 30 min. The wells were washed, mounted and observed under a fluorescent microscope.

### Statistical analysis

All assays were performed in triplicate and the results were analyzed statistically (standard error and ANOVA) using Microsoft Office Excel 2010 and one-way ANOVA calculator.

## RESULTS

### Hydrogen peroxide assay

**Figure 1.**
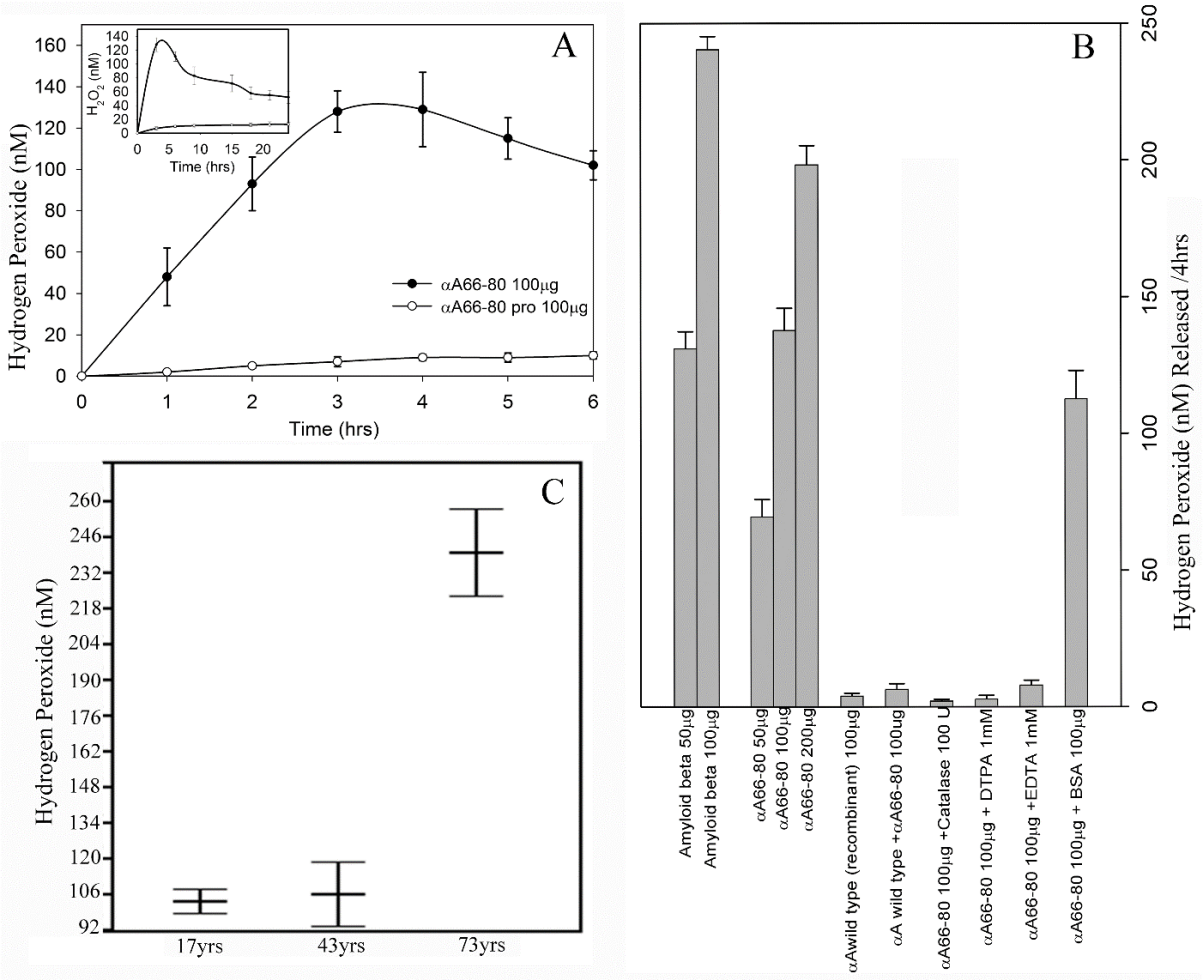
**Hydrogen peroxide generation by crystallin derived peptides and β-amyloid peptide. A)** Generation of H_2_O_2_ by αA66-80 peptide. αA66-80 peptide (1mg/mL) was incubated in 50mM phosphate buffer for different durations, up to 24 h. The sample was withdrawn every hour and H_2_O_2_ generation was monitored using Amplex red reagent. Closed circle, αA66-80 peptide; open circle, αA66-80pro. Inset shows the generation of H_2_O_2_ up to 24 h (avarage of three experiments). **B)** Relative amount of H_2_O_2_ generation by αA66-80 peptide, β-amyloid and α-crystallin. Assays were carried out in 50 mM phosphate buffer. H_2_O_2_ generation is presented in nano Molar scale. Data shown is the avarage of three independent experiments. H_2_O_2_ generation by αA66-80 peptide in the presence or absence of αA-crystallin or metal ion chelators or catalase is also shown in the figure. **C)** Comparision of H_2_O_2_ generation by LMW peptides isolated from 73-, 43- and 17-year-old human lenses. LMW peptides were isolated and assayed as described in methods section. The data obtained by Amplex red assay show a greater amount of H_2_O_2_ generation from LMW peptides of 73-year-old lenses. (The results shown are an avarage of three independent experiments.)

αA66-80 peptide-mediated H_2_O_2_ generation was measured using Amplex Red assay kit. The kit uses 10-acetyl-3,7-dihydroxyphenoxazine, which readily reacts with H_2_O_2_ and produces a red-fluorescent oxidant product, resorufin, that can be measured by fluorometry. The peptide αA66-80 (1mg/mL) was prepared in phosphate buffer and incubated at 37°C for different durations (0-24 h) to examine the ability of the αA66-80 peptide to catalyse the formation of hydrogen peroxide. Fig. 1A shows the time-dependent increase in the generation of H_2_O_2_ by the peptide up to 3 h. The samples that contained 100 µg of the peptide and incubated for 1 h in 100 µl phosphate buffer released 45±11 nM of H_2_O_2_. The maximum amount of H_2_O_2_ generation occurred following incubation for 3 h to 4 h. Incubation beyond 4 h to 24 h generated relatively less detectable H_2_O_2_ (Fig. 1A inset) and the H_2_O_2_ present in the reaction mixture started to degrade. Proline-substituted αA66-80 peptide containing sample yielded negligible fluorescence, even after an extended duration of incubation. Based on the data, an incubation period of 4 h was used to evaluate H_2_O_2_ generation from αA66-80 peptide under different conditions. In other experiments, we incubated the αA66-80 peptide at concentrations of 50, 100 and 200 µg in 100 µl phosphate buffer. Figure 1B shows the concentration-dependent generation of H_2_O_2_ by αA66-80 peptide, β-amyloid peptide and αA-crystallin. The αA66-80 peptide generated about 51% less H_2_O_2_ than β-amyloid peptide (Fig. 1B). Under the same incubation conditions, αA66-80 peptide (50 µg) generated 68 ± 9 nM H_2_O_2_ in 4 h, whereas Aβ peptide (50 µg) generated 131 ± 8 nM H_2_O_2_, indicating that fibril-forming peptides have varying ability to generate H_2_O_2_. In another set of experiments, αA-crystallin itself (100 µg), incubated in 100 µl phosphate buffer for 4 h, showed only a negligible amount of H_2_O_2_ generation. Co-incubation of αA-crystallin (100 µg) and αA66-80 (100 µg) resulted in suppression of H_2_O_2_ generation by the peptide, whereas the addition of bovine serum albumin (100 µg) during the incubation of αA66-80 peptide (100 µg) led to a marginal decrease in peptide-induced H_2_O_2_ generation. Together, the results indicate that αA-crystallin has the ability to suppress H_2_O_2_ generation by αA66-80 peptide.

### Generation of H_2_O_2_ from total LMW peptides obtained from human lens

To investigate whether peptides present in human lenses have the ability to generate H_2_O_2_, we isolated LMW peptide from the total urea soluble lens extracts of 17-year-old, 43-year-old and 73-year-old lenses using a 10 kD cut off membrane filter. There was a significantly higher amount of H_2_O_2_ generation (240 ±17nM) from 100 µg of native LMW peptides from 73-year-old lens extracts as compared to that found in 43-year-old lens extracts (107 ±13 nM) and 17-year-old lens extracts (103 ±5 nM) (Fig. 1C). The same amount (100 µg) of synthetic αA66-80 peptide generated only 127 ± 12 nM H_2_O_2_ during the same incubation period, indicating that the peptides generated from post-translationally modified proteins may have a greater ability to generate H_2_O_2_. It is known that aged lenses have several post-translationally modified proteins and peptides. Additionally, there might be several peptides that have a greater ability to generate H_2_O_2_ than αA66-80 peptide, or H_2_O_2_ generation could represent an additive effect of the mixture of peptides. A separate study is required to characterize each of the endogenous peptides present in aged lens tissues for their ability to form fibrils and generate H_2_O_2_. It should be noted that the total water-soluble lens proteins, or isolated α-crystallin or other lens crystallin fractions or recombinant αA-crystallin generated significantly lower amounts of H_2_O_2_ than did αA66-80 peptide (Table 1 and Fig. 1B).

Generation of H_2_O_2_ from trypsin-digested crystallin fragments in vitro

From the present study we know that the endogenous peptide αA66-80 and lens LMW peptides generate hydrogen peroxide in vitro whereas the full-length αA-crystallin does negligible amounts. To test whether trypsin digested lens crystallin fragments generate H_2_O_2_, we incubated 100 µg of peptides prepared from digesting α, βH, βL and γ-crystallins (obtained from 40- to 43- year-old lenses) in phosphate buffer for 4 h and measured the released H_2_O_2_. Table 1 highlights the data on H_2_O_2_ generation by peptides formed from different crystallin fractions. The results show that tryptic peptides obtained from α-, βH-, βL- and γ-crystallins generate significantly higher amounts of H_2_O_2_ compared to the parent proteins. The trypsin-digested α-crystallin fraction showed 208 ± 6.4 nM H_2_O_2_ generation, whereas the same amount of whole crystallin generated only 11.3 ± 1.2 nM of H_2_O_2_ after 4 h of incubation. Similar differences were observed between βH-, βL- and γ-crystallins and peptides derived from them. As a control, we used BSA, either undigested or trypsin-digested, and found that neither BSA nor the trypsin-digested BSA generated a significant amount of H_2_O_2_, indicating that the generation of H_2_O_2_ by peptides is selective. Further, the data also reveal that tryptic fragments of endogenous lens α-crystallin generate more H_2_O_2_ than the tryptic fragment of recombinant αA-crystallin, indicating that peptides prepared from endogenous crystallins have an enhanced capacity to generate H_2_O_2_.

**Table 1:** Hydrogen peroxide generation by lens crystallin fractions and crystallin-derived peptides*

Proteins / peptides (100 µg)	H_2_O_2_ generation (nM)
Total lens extract (43-45yrs.)	5.7 + 0.9
α-crystallin fraction	11.3 + 1.2
βH-crystallin fraction	6.1 + 0.2
βL-crystallin fraction	7.5 + 0.2
γ-crystallin fraction	6.7 + 0.3
Recombinant αA-crystallin	0.5 + 0.0
BSA	0.2 + 0.0
LMW peptides from trypsin-digested α-fraction	208.1 + 6.4
LMW peptides from trypsin-digested βH -fraction	156.2 + 6.2
LMW peptides from trypsin-digested βL -fraction	184.4 + 7.9
LMW peptides from trypsin-digested γ -fraction	150.0 + 4.3
LMW peptides from trypsin-digested αA-crystallin (recombinant)	29.8 + 0.2
LMW peptides from trypsin-digested BSA	3.2 + 0.0
αA66-80	127 ± 12
αA66-80 (V72P)	4.5 + 0.3
αA66-80 (H79A)	1.9+ 0.2

* Human lens crystallin fractions and crystallin-derived low molecular weight (LMW) peptides (100 µg) were incubated in phosphate buffer at 37°C for 4 h. At the end of the incubation, the amount of H_2_O_2_ formed was estimated, as described in the methods section. The amount of generated H_2_O_2_ is the average of three independent measurements.

### Metal binding to the peptide, chelator, and catalase effect on the generation of H_2_O_2_ by αA66-80 peptide

**Figure 2.**
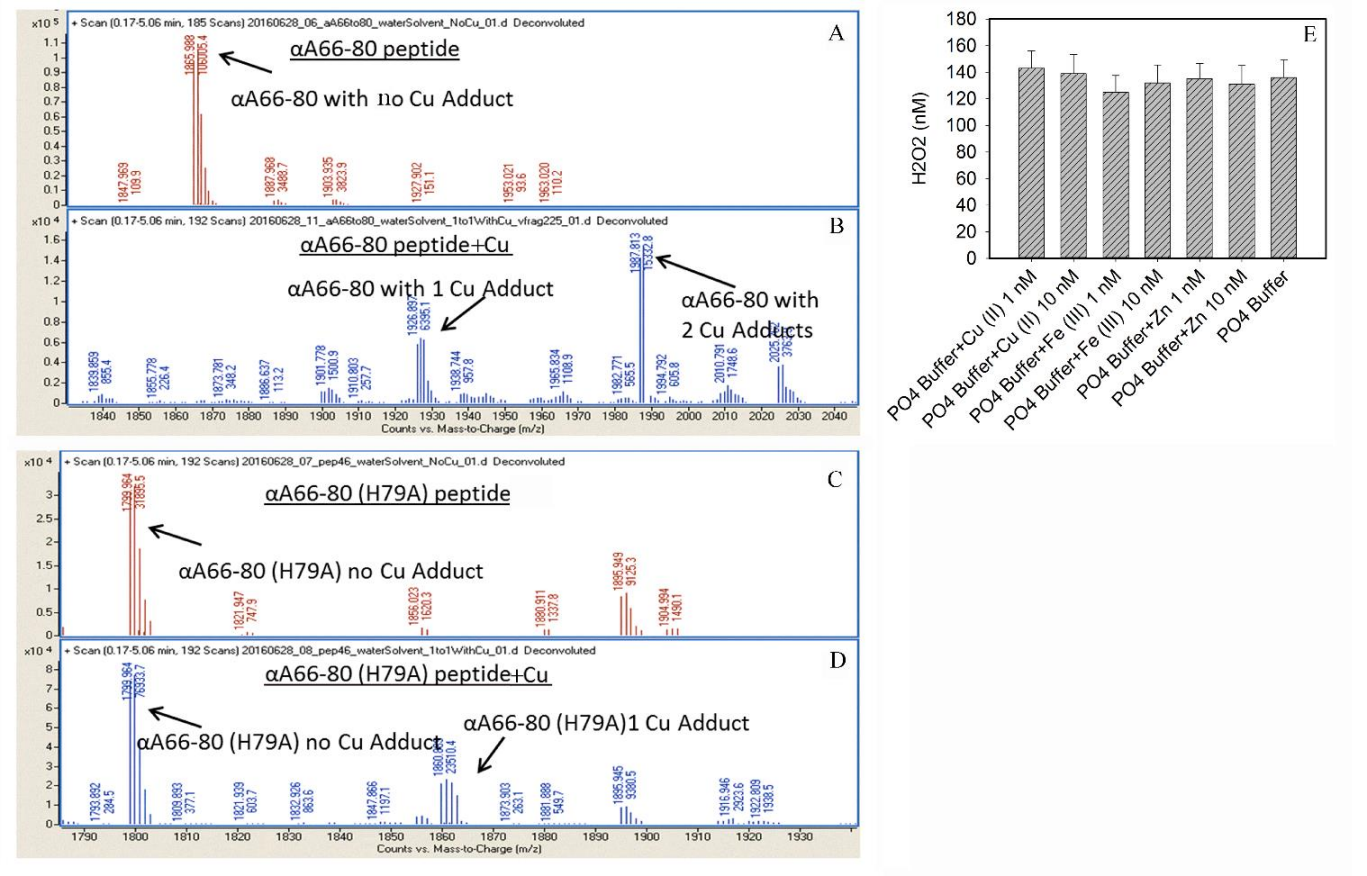
**A-D Nanosprary QTOF spectra of peptides with and without copper in aqueous solution. A)** Peptide αA66-80, **B)** Peptide αA66-80 in presence of excess copper sulphate, **C)** Peptide αA66-80 (H79A), **D)** Peptide αA66-80 (H79A) in presence of excess copper sulphate. The αA66-80 peptide can bind upto 2 copper (peak at 1986.81 Da). The two-copper binding peak is suppressed in αA66-80 H79A peptide. The results suggest histidine role in copper binding and subsequent H_2_O_2_ generation by αA66-80 peptide. **E)** Generation of H_2_O_2_ by αA66-80 peptide in presence of different metal ions added to 50 mM Phoshpahte buffer, pH 7.2. To test whether addition of metal ions can increase the genration H_2_O_2_ by αA66-80 peptide, we incubated the peptide either alone or with Cu (11), Fe (III) or Zn(II), in 1 nM or 10 nM, for 4 hr in PO4 buffer. At the end of incubation, Amplex Red reagent was added and the mixtures were further incubated for 30 min in the dark at room temperature. The fluorescent intensities of the reaction mixtures were measured as mentioned in the method.

The binding of metal ion (copper) to αA66-80 peptide was confirmed by mass spectrometry. We found that αA66-80 peptide binds up to two copper ions whereas the histidine replaced peptide (αA66-80H79A) binds only one copper ion suggesting that the histidine residue in the peptide plays a critical role in metal binding (Fig. 2A-D). Metal ion is important in the oxidation process. Since we used phosphate buffer prepared in deionized water, we thought that the addition of metal ion to the phosphate buffer would increase the generation of H_2_O_2_. Therefore, we carried out a series of experiments in which the αA66-80 peptide was incubated either alone or with Cu (11), Fe (III) or Zn (II), in 1 nM or 10 nM, for 4 h in phosphate buffer. The results revealed no significant difference in the level of H_2_O_2_ generation by the αA66-80 peptide when it was incubated alone or with the added metal ion (Fig. 2E), which raised the question of whether the phosphate buffer used in this study was contaminated with metal ions. Analysis of the phosphate buffer by flame photometry showed the presence of 0.021 ng/mL Cu (II) and 0.18 ng/mL Mg (II). The data suggest that the trace amount of metal ions present in the phosphate buffer itself is sufficient to form a redox center in the peptide and generate H_2_O_2_. Further, to demonstrate the presence of metal ion in the phosphate buffer, we treated the phosphate buffer with Chelex 100 before beginning the experiments with αA66-80 peptide. Pretreatment of phosphate buffer with the chelating agent stopped the generation of H_2_O_2_, indicating that the metal ion present in phosphate buffer was responsible for the H_2_O_2_ formation. In other experiments, we incubated αA66-80 peptide in the phosphate buffer containing 1mM ethylenediaminetetraacetic acid (EDTA) or 1mM diethylenetriamine pentaacetic acid (DTPA) and found complete abolition of H_2_O_2_ formation, confirming that the trace metal ion in the phosphate buffer was required for the generation of H_2_O_2_ by the αA66-80 peptide. To investigate further, we incubated αA66-80 peptide in phosphate buffer in the presence of catalase, an enzyme that decomposes hydrogen peroxide to water and oxygen. As we expected, the αA66-80 peptide incubated with catalase produced no detectable H_2_O_2_. Though αA66-80 has the ability to bind crystallins and other proteins and impair biological function, the peptide does not, interestingly, modify catalase enzyme activity.

**Figure 3.**
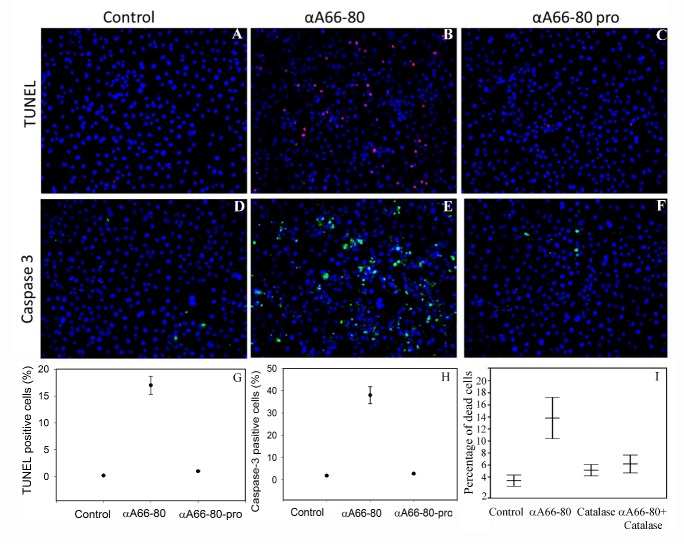
**αA66-80 peptide--induced cell apoptosis and caspase-3.** Peptide-induced cell apoptosis was assessed in the presence and absence of αA66-80 peptide or proline-substituted peptide (αA66-80 pro). **A-C** and **G**) TUNEL assay, in which the blue stain shows intact nucleus and the red stain indicates fragmented DNA inside the nucleus indicating cell apoptosis. **D-F** and **H**) Caspase-3 assay, in which the blue stain shows the nucleus and the green stain shows cleaved caspase-3 antibody reactivity. Note that most of the green stain is in the cytoplasmic region. **I)** Effect of catalase on Cos-7 cells treated with αA66-80 peptide. Cos-7 cells were cultured and treated as described under methods. Cells were stained with live/dead cell staining according to the EarlyTox live/dead cell potocol using Spectra Max i3 plate reader. αA66-80 (10µg); Catalase (500 units) and αA66-80(10µg) +Catalase (500 units) (The result shown are the avarage of three independent experiments.)

### αA66-80 peptide induces cell apoptosis by generating H_2_O_2_

To investigate whether the H_2_O_2_ produced by αA66-80 induces cell apoptosis, similar to exogenously supplied H_2_O_2_, Cos-7 cells were treated with αA66-80 peptide for 4 h in serum-free media. TUNEL assay after 24 h of incubation demonstrated that about 15% of the cells were TUNEL positive (Fig. 3 B and G). There was a higher percentage of apoptotic cells in the αA66-80-treated cultures compared to that observed in our previous study in which cultures were treated with 150 µM H_2_O_2_ [38], suggesting that αA66-80-mediated cell apoptosis might result from the combined effect of αA66-80 peptide-induced protein aggregation [18] and peptide-mediated H_2_O_2_ generation. Under similar assay conditions, proline-substituted αA66-80 treated cells or control cells did not show much DNA fragmentation (Fig. 3 A, C and G). The Cos-7 cells treated with αA66-80 peptide (10µg) in presence of catalase (500 unit) did not display a significant increase in apoptosis (Fig. 3I) suggesting a role for H_2_O_2_ generated from the peptide in apoptosis.

**Figure 4.**
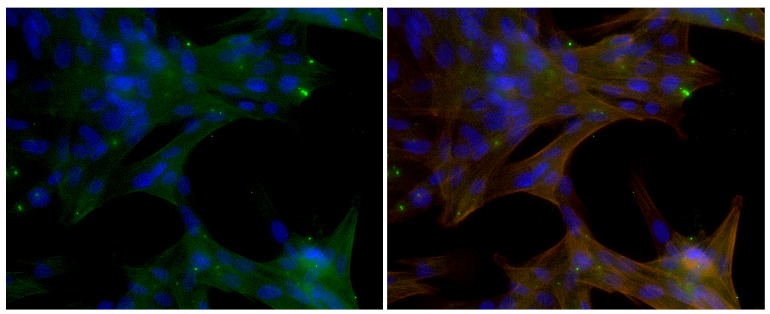
**Entry of αA66-80 into cells.** Pig primary LECs were treated with αA66-80-FITC for 24 h and the entry of fluorescent peptide (green) into the cells was visualized by observing the cells under fluorescent microscope after counter staining with DAPI (blue - nucleus) and Phalloidin (red - actin). Left image shows the composite of blue and green channels and the right image is a composite of all three channels.

### αA66-80-induced apoptosis occurs by caspase activation

Activation of caspases, a family of cysteine proteases, is one of the central events in the apoptotic pathway. During the apoptotic process pro-caspase-3 is cleaved to active caspase-3 [39]. The cleaved form of caspase-3 can be detected by the monoclonal antibody. To test whether the cell apoptosis induced by αA66-80 is following a caspase-dependent pathway, we used an in situ antibody staining protocol, as described in the methods section. TUNEL-positive cells and expression of cleaved caspase-3 were detected in the αA66-80-treated culture. Whereas no TUNEL positive cells and no expression of cleaved caspase-3 were detected in proline-substituted αA66-80 treated cells or in control cells (Fig. 3 D, F and H), providing evidence of αA66-80-mediated apoptosis through caspase-3 activation.

**Figure 5.**
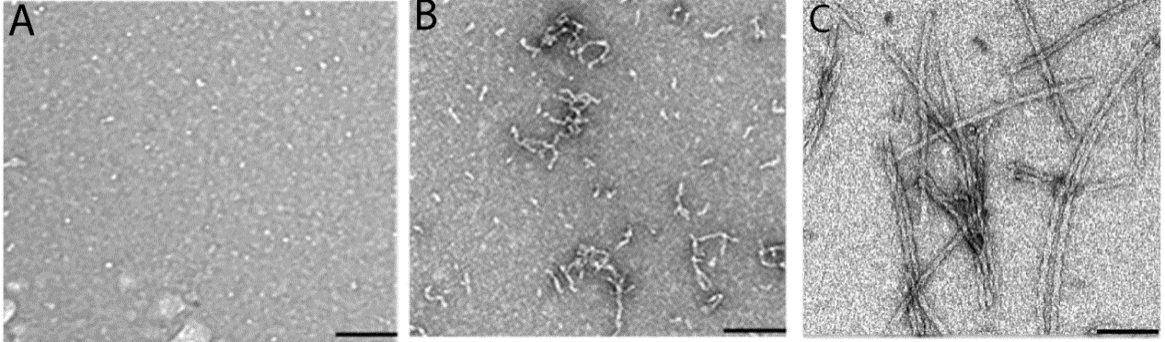
**Peptide αA66-80 forms amyloid-like fibrils.** The peptide αA66-80 was incubated in phosphate buffer at 37°C. The samples were withdrawn at different time intervals (0 hr, 4 hr and 24 hr) and the fibril-like structure of αA66-80 peptides was visualized and recorded under TEM. A) 0 hr; B) 4 h; and C) 24 h incubations. The scale bar is 100 µm.

### αA66-80 peptide can enter cells without the aid of delivery agents

To assess if αA66-80 peptide can enter the cells to exhibit apoptotic activity we have used FITC conjugated peptide and looked for its presence in cells. Green signal for FITC was detected inside pig LECs at 24 h suggesting the entry of peptide into cells without the aid of delivery agents (Fig 4). Majority of the peptide that enter the cells gets co-localized with cytoskeletal protein, actin. Some aggregates of αA66-80 peptide were also seen which appeared to be on the outside the cell

## DISCUSSION

Functionally impaired proteins and their fragments, under some conditions, form highly ordered fibrillar aggregates and the accumulation of these fibrils gives rise to pathological conditions, including neurodegenerative disease and cataract [40]. Several fibril-forming peptides, such as Aβ peptide in Alzheimer’s disease [41, 42], islet amyloid peptide in type II diabetes [43], and fibrinogen α-chain peptides in fibrinogen amyloidosis [44], have been linked to many pathological conditions. In our previous studies to understand the role of LMW peptides in cataractogenesis, we identified, for the first time, a 15 amino acid length endogenous peptide, αA66-80, derived from of αA-crystallin that accumulates in aged and cataract lenses. Our pioneering studies of αA66-80 provide solid evidence that this peptide has a role in age-related cataract formation: αA66-80 promotes protein aggregation and causes light scattering protein aggregates to be formed in vitro, similar to aggregates found in cataractous lenses [19, 20]. Further, we showed that the αA66-80 peptide possesses a well-defined beta sheet signature, as demonstrated by far-ultraviolet (UV) CD profile, and an unbranched fibril structure, as seen under transmission electron microscopy (TEM) (Fig. 5). In addition, the peptide interacts with Thioflavin T and Congo red dyes, which are known to bind to amyloid fibrils [45]. Therefore, the principal properties of the αA66-80 peptide mirror the characteristics of β-amyloid peptide. It is also interesting that the αA66-80 peptide possesses a core sequence, _70_KFVIF_74_, that closely resembles the Aβ region _16_KLVFF_20_ which is believed to be responsible for fibril formation [46, 47]. Because of the similarities in properties between the Aβ peptide and αA66-80 peptide, we under took the present study to test whether the αA66-80 peptide, like Aβ peptide, has the ability to generate hydrogen peroxide. The results clearly demonstrate the generation of H_2_O_2_ from αA66-80 peptide (Fig 1A&B). The maximal αA66-80-mediated generation of H_2_O_2_ was observed in the first 3 to 4 h of incubation. TEM analysis of αA66-80 peptide incubated for 4 h showed only proto fibrils (Fig. 5), without any mature fibrils, indicating that H_2_O_2_ generation takes place during protofibril assembly. This observation is consistent with the study by Tabner et al, who reported that Aβ 1-40 generates H_2_O_2_ as a short burst during the early incubation period, when mature amyloid fibrils are yet to be formed [48]. Similar observations have been reported by others studying Aβ peptide [49, 50]. Further, we observed that αA66-80 peptide generates about 51% less H_2_O_2_ than Aβ peptide, indicating that the various peptides that form fibrils might have different capacities to generate H_2_O_2._

Metal ions play a vital role in redox activity. Studies have shown that the Aβ peptide and other amyloidogenic peptides bind metal ions to form a peptide-metal ion complex that acts as a radical-generating system believed to be responsible for amyloid-mediated cell toxicity [51,58]. The copper-dependent hydrogen peroxide generation via the electron transfer reactions similar to that shown below is likely the mechanism of generation of H_2_O_2_ by αA66-80 peptide. The failure to

$$\begin{array}{l} \alpha A66\text{−}80\text{−−−Cu}(I)+O_{2}\to \alpha A66\text{−}80\text{−−−Cu}(II)+{O_{2}}^{.-}\\ O2^{.-}+e+2H+\to H_{2}O_{2} \end{array}$$

generate H_2_O_2_ by the peptide in the presence of metal ion chelators supports this view.

Because the αA66-80 peptide forms amyloid fibrils, we investigated whether it has the ability to bind metal ion, thereby making the peptide a free radical-generating system. From our previous studies we know that the αA70-88 sequence binds Cu (II) [35]. Interestingly, the Cu (II) binding region in αA70-88 peptide has an overlapping region with the αA66-80 peptide, leading us to strongly believe that the αA66-80 peptide binds Cu (II). Since we know that phosphate buffer contains a trace amount of Cu (II), we incubated the αA66-80 peptide in the phosphate buffer with 1mM EDTA and observed that the reaction mixture does not generate H_2_O_2_, confirming that chelation of metal ion prevents the formation of peptide-metal ion redox system. When we used Chelex-100-treated phosphate buffer to incubate the αA66-80 peptide, there was no H_2_O_2_ generation, indicating that H_2_O_2_ is formed by αA66-80 in a metal ion-dependent manner. The αA66-80 H79A peptide that showed significant reduction in Cu (II) binding, based on mass spectromentric analysis (Fig. 2 A-D) showed >98 percent reduction in H_2_O_2_ generation indicating that metal binding via histidine is required for maximal generation of H_2_O_2_. Furthermore, H_2_O_2_ production by the αA66-80 V79P peptide was also reduced that does not form fibrils [57] suggests that the ability to form fibrils is also essential for the creation of redox center responsible for H_2_O_2_ generation. Additional studies is required to determine how the fibril formation property bring together the amino acids involved in metal ion binding to achieve the necessary co-ordination.

In an earlier study we showed that aged and cataract lenses accumulate several endogenous peptides derived from crystallins, including αA66-80 peptide [19]. More than 200 LMW peptides have been identified in lens tissue [21], including the 25 highly abundant LMW peptides we previously identified in our lab [19]. Several other crystallin fragments similar to the αA66-80 peptide are also likely to be involved in the generation of H_2_O_2_ in lenses_._ In support of this view, we found that the LMW peptide fraction isolated from human lens tissue generates H_2_O_2_ and that <10 kDa peptides from 73-year-old human lenses produce 2-fold more H_2_O_2_ than <10 kDa peptides from 17- and 43-year-old human lenses (Fig. 1C). The data point to the existence of several other peptides in aged human lenses with the ability to generate H_2_O_2._ We have observed a yellowish brown color in the LMW peptides isolated from 73-year-old lenses as compared to the clear appearance of peptides from young lenses, indicating that extensive post-translational modification has occurred in peptides of aged lenses. The coloration is likely due to the presence of advanced glycation end-products (AGEs) [52], which might also be a contributing factor in the increased H_2_O_2_ generation by peptides of aged lenses. Further, a series of experiments to determine whether enzymatically digested crystallins generate H_2_O_2_ showed that, while the crystallin proteins by themselves do not generate significant amounts of H_2_O_2,_ the trypsin-digested αA-, βH-, βL- and γ-crystallin fragments do generate H_2_O_2_ (Table 1). To discern whether peptides from non-lens proteins generate H_2_O_2_ or whether H_2_O_2_ generation is a specific characteristic of αA-crystallin fragments, we conducted a set of experiments using peptides derived from bovine serum albumin (BSA) and found that both native and trypsin-digested BSA generated only negligible amounts of H_2_O_2_ compared to crystallin fragments. Although α-crystallin is known to suppress bound metal ion-mediated H_2_O_2_ production [33, 34], the present study shows that peptides released from the α-crystallin generate H_2_O_2_ in the presence of metal ions. Further studies are needed to determine the full impact of this novel mechanism of peptide-mediated H_2_O_2_ *in vivo*.

It is well known that the accumulation of endogenous peptide is the net result of proteolysis [53, 54]. Since the endogenously accumulated peptide generates H_2_O_2_ and exerts cell apoptosis [36], we investigated whether exogenously transduced synthetic peptide αA66-80 can enter the cell and induce cell apoptosis. The experiments carried out with labelled peptide clearly indicates that αA66-80 can enter the cells without the aid of delivery agents. A major part of the peptides transduced into the cells interact with cytoskeletal proteins. The impact of such in vivo interactions on the H_2_O_2_ generation and toxicity by αA66-80 is not clear. We have observed that binding of αA66-80 peptide to α-crystallin decreases the amount of H_2_O_2_ generation by the peptide in vitro. We observed that production of H_2_O_2_ by αA66-80 peptide is rapid and peaks at 4 h which might be sufficient to overwhelm the cellular defense mechanisms and trigger apoptotic pathways. After triggering apoptotic response if the peptide interacts with other cellular proteins it may not affect the H_2_O_2_-induced toxic effect of the peptide. Our fluorescence staining experiments with labelled αA66-80 peptide was done 24 h after treating the peptide. Further experiments are required to see if the binding of peptide to cellular proteins occurs within that short-burst period of H_2_O_2_ production and to delineate the cytotoxic action of αA66-80 peptide. Cos-7 cells were selected to see if the transduced peptide can induce apoptosis, and two different assays were used to assess apoptosis: (1) TUNEL assay, to see the fragmented DNA in the cell nuclei, the target event in the apoptosis signaling pathway, and (2) caspase-3 assay, since caspase activation is a central event in the apoptotic pathway, in which pro-caspase-3 cleaves to active caspase-3 to begin the progression of the apoptotic pathway [55]. The experimental results revealed that αA66-80 peptide-transduced cells are positive for TUNEL, but those cells treated with control peptide, proline-substituted αA66-80 peptide do not show a similar amount of TUNEL-positive cells (Fig. 3), a sign of apoptotic cell death. Consistent with the TUNEL assay results, caspase-3 antibody signals were strong only in αA66-80-treated cells. We previously reported that 150 µM of H_2_O_2_ induces roughly 10% TUNEL-positive cells in Cos7 cells [38], whereas in the present study, though we observed a nano-molar range of H_2_O_2_ generation by αA66-80, we found more than 15% TUNEL-positive cells, indicating that the peptide-mediated cell death may be associated with the combined effect of H_2_O_2_ generation and peptide-induced protein aggregation. Throughout this series of experiments, we used a higher amount of αA66-80 peptide than the amount of endogenously available peptide in lens tissues in order to visualize the difference in a reasonable time period. Although intact α-crystallin suppresses about 95% of H_2_O_2_ generation by αA66-80 peptide in vitro, given the longevity of the lens tissue, we have taken excess amount of peptide in our studies and also believe that the cumulative effect of H_2_O_2_ on lens proteins would be significant. In support of this is the observation that nuclear opacity develops in HBO-treated guinea pig lenses in the presence of a 2-fold increase in the lens oxygen level [56]. Additionally, we have shown that the HBO-treated guinea pig lenses have an increased level of αA66-80 peptide compared to age-matched non-HBO-treated controls [20], suggesting that the αA66-80 peptide has a role in the oxidation of lens crystallins and the development of nuclear opacity in HBO-treated lenses. Although many properties of the αA66-80 peptide are now known, such as its propensity for protein aggregation, the formation of amyloid fibril-like structures and the generation of H_2_O_2_, the full spectrum of the effect of αA66-80 peptide in vivo is yet to be understood.

In summary, the endogenous peptide αA66-80 acts as a redox center and generates hydrogen peroxide. A chelator such as EDTA blocks the formation of H_2_O_2_, indicating that the release of hydrogen peroxide by αA66-80 peptide is metal ion-dependent. The αA66-80 peptide is also capable of inducing cell apoptosis in Cos7 cells. Our study suggests that the hydrogen peroxide generated from the αA66-80 peptide in vivo could be responsible for some of the oxidative damage seen in aged and cataract lenses.
